# Using Inquiry-Based Learning to Enhance Immunology Laboratory Skills

**DOI:** 10.3389/fimmu.2019.02510

**Published:** 2019-10-22

**Authors:** Maria Demaria, Anita Barry, Kim Murphy

**Affiliations:** Department of Immunology and Pathology, Monash University, Melbourne, VIC, Australia

**Keywords:** inflammation, undergraduate student, pedagogy, inquiry-based learning, active learning and teaching methodologies, laboratory skills

## Abstract

A challenge in teaching immunology in the undergraduate laboratory is to encompass the many varied skills that need to be applied when performing an investigative study of such a complex area. It requires background knowledge, data analysis skills, critical thinking, and design capacities to include relevant controls and applications of particular techniques to answer a research question. It also requires strong technical skills. One such approach is to use inquiry-based learning which allows students a more proactive and integrative role in their learning. In one of our final year immunology units we have incorporated an inquiry-based exercise that runs across four 5-hour sessions. Students are given two cornerstone immunology techniques (ELISA and a flow cytometry-based cytokine bead array), which they use to formulate a study investigating inflammation. Stage one is to design the experiment with some guidance from teaching staff, stage two is to perform the experiment, and then finally students are required to analyze the data, apply appropriate statistics, and write a report outlining their findings. This approach provides students ownership of the process and allows them the opportunity to investigate a real-world problem rather than just attempting to obtain the expected “correct answer.” Feedback from both students and staff has been positive with strong engagement and high quality reports produced.

## Introduction

As a discipline, immunology is considered a difficult area to master by many undergraduate students (1, 2). Using traditional undergraduate teaching strategies students learn many of the basic techniques fundamental to immunology. However, to create innovative learners, it is necessary to move from the recipe-based approach that is common in laboratory classes to a more creative mode of teaching. We use a mix of traditional, recipe-based laboratory classes, and open-inquiry based approaches to enhance creativity and scientific knowledge in our students.

By their very nature, practical classes are active learning environments. Active-learning approaches encompass many different teaching activities, but are designed to have students actively engaged in their own learning. One style of active learning is inquiry-based learning. Active learning in general, and inquiry-based learning in particular, has been reported to improve student scientific literacy (3), improve student retention (4), and reduce failure rates (5). A difficulty in assessing the effectiveness of inquiry-based learning is that there is no clear definition, and different researchers use the same terminology for different activities (6).

Generally, inquiry-based learning approaches are student-centered learning activities that require student immersion in the learning process. Banchi and Bell (7), as well as others (6, 8) describe an “inquiry-continuum,” from highly structured with a large amount of input and direction from facilitators (sometimes known as confirmation inquiry) to open-inquiry, whereby students develop a question for testing, a method or procedure to address the question, and develop a solution to the question. Here we focus on research that has used genuine inquiry, whereby students are expected to search for an answer to a problem and construct their own knowledge, rather than other problem-based learning approaches where students are often seeking the “right” answer.

Inquiry-based learning is difficult to implement, particularly at the university-level and in large classes, but Summerlee and Murray (9) used a longitudinal study to determine the longer-term advantages of inquiry-based learning techniques. In this study, students from diverse university courses were followed over the length of their studies. Students with the lowest grades on entering university and who engaged in inquiry-based learning in their first year showed the greatest improvement in their final year marks compared to the highest-achieving students, suggesting the inquiry-based learning approach assisted students to become more engaged in learning, and more confident in accessing research resources to assist their learning. In a study by Spronken-Smith and Walker (10), investigating different levels of inquiry-based learning, classes that used the most open inquiry level tasks had the best outcomes in terms of students understanding the scientific process within the particular discipline being studied. In another study, by Lord and Orkwiszewski (11), students completed laboratory classes in either a traditional directed or inquiry-based manner. Students in the inquiry based group not only demonstrated increased understanding of discipline knowledge, they also enhanced their scientific thinking skills and appreciation of the scientific process.

Inquiry-based learning does place a strain on students, requiring a high-cognitive load, and some educators believe that this approach is a less efficient and less effective form of instruction (12). However, the skills that science students can learn from this approach include scientific thinking and research processes. Not every practical class should be based around inquiry-based learning, as the basic skills taught in more traditional laboratory classes underpin necessary skills for the inquiry-based tasks (13), and using just inquiry-based methods can create frustrations for students (3). However, the higher level thinking that inquiry requires means that incorporating it at some stage of the curriculum can help students develop skills that are transferable to discipline-based research (3, 14).

There are few examples in the literature on methods to successfully integrate inquiry based learning for teaching immunology laboratory practical skills in undergraduate laboratories. Manzoni-De-Almeida et al. (15) describe a guided-inquiry approach to develop immunology-specific knowledge in undergraduate and graduate laboratories. They found, even using a quite structured approach to inquiry, that students improved their scientific processes knowledge and improved the current (or future) links between students and researchers. Another paper, from Gunn et al. (16), describes the design of a module for level 2 inquiry-based learning (structured) looking at the molecular outcomes of a range of mediators of inflammation. This module was aimed at students earlier in their course progression than what has been implemented for our course. Finally, Berkes and Chan (17) describe an undergraduate immunology project that includes inquiry-based tasks to develop hypotheses and test the effect of a range of anti-inflammatories on macrophage cytokine production. Despite these inquiry-based tasks only forming part of the wider project, upon completion students still demonstrated enhanced confidence and awareness of both the scientific process and also immunology-specific laboratory skills. Here, we describe one example of a more open level of inquiry for students further progressed in their degree and closer to starting employment or a postgraduate degree; the described activity has not been formally evaluated, and is offered as an example activity that others may replicate in their teaching. We have drawn on the described published insights that inquiry-based learning can be beneficial in the right context and applied these when developing this exercise. The unit in which this exercise is performed is practical-based, that also has other more traditional style practical classes covering aspects of immunology such as allergy, diagnostic techniques for rheumatic diseases and influenza testing to strike a balance between delivery methods. The exercise also builds on previously learnt knowledge in the degree as level 2 immunology is a pre-requisite.

## Implementing an Inquiry-Based Practical into the Curriculum

We feel that incorporating a genuine inquiry-based practical as part of our curriculum is an important pedagogical approach that helps students to develop relevant general scientific and research-discipline specific skills, as well as achieving key graduate attributes of becoming critical and creative scholars (18). In our curriculum, inquiry-based learning is defined by a student-designed experimental approach with a genuine creation of new knowledge. Students, and therefore educators, aren't seeking the “right” result, rather they are developing and applying scientific thinking and principles to their work.

Students that complete an immunology major at our university complete two level 2 units and four level 3 units. At level three students have a choice of two theory units, and three practical units to fulfill the requirement of the four units. At the completion of the major, students are expected to have high-level immunology knowledge as well as demonstrate a deep understanding of the scientific process and how to design and evaluate methods to investigate immunological problems. These units use a variety of teaching and learning approaches, with numerous opportunities to be actively engaged in the generation of knowledge. In one of our immunology level 3 practical units, we use an inquiry-based learning technique. Students develop a research question, design a methodology to address the question, and then write a report on their work. Students test their own saliva samples (at two time points) for the presence of a number of inflammatory (and anti-inflammatory) markers through the use of a competitive ELISA (to test levels of LTB4) and cytokine bead array (CBA; to test for IL-1β, IL-6, IL-8, IL-10, IL-12p70, and TNF). These two techniques cover a range of pro- and anti-inflammatory molecules, allowing for a more realistic and detailed analysis of the inflammatory response to the chosen stimuli. Students approached this assessment in four major steps; background research, research design, conducting the experiment, and reporting.

In the first stage, students are introduced to the idea of testing their saliva for the presence of inflammatory markers and techniques used for detection and quantification (competitive ELISA and CBA). Student groups then brainstorm ideas that may impact inflammation; they are encouraged to reflect on their knowledge from previous units to identify relevant ideas, and additionally draw on ideas that are presented in the popular media, or from cultural backgrounds. Ideas range from consuming green tea or turmeric lattes, to undertaking vigorous exercise; generally groups brainstorm 20–25 topics. Within each group, students are assigned a few topics to research in more depth; students are reminded to use databases such as PubMed to ascertain the availability, or lack, of current evidence regarding the role of their proposed idea in influencing inflammation, as well as other at factors that impact any influence (e.g., dose or timing). This initial reading and investigation supports skills and graduate attributes in accessing and evaluating appropriate resources.

Following their background research, students discuss their literature findings in the next session with their teaching associate (TA) and student group. Students then choose an intervention, discuss, and agree on a design including appropriate data analysis. Most chosen interventions are based on consuming either a particular food or drink (e.g., eating boiled peanuts daily for 1 week, with saliva samples collected on day 0 and 7; drinking one shot of tequila, with saliva samples collected before alcohol consumption and 30 min after consumption), although engaging in exercise has also proven popular. This stage of the inquiry-based design encourages and supports scientific thinking, including generating a hypothesis, and data analysis. Students are also supported to identify potentially confounding variables, appropriate controls, and discuss ethics. For example, the group of students who chose to investigate the proposed anti-inflammatory effects of tequila needed to discuss the ethics of consuming alcohol before class and how they could alleviate the effects, deciding on consuming a meal. Here, students opted to consume a pre-determined, healthy meal, to mitigate the confounding effects of food on the inflammatory response.

Following the design stage, student groups (*n* = 8–12) perform their chosen intervention, collect their saliva samples and then perform their competitive ELISA and CBA. These techniques, while using quite advanced skills, still use a traditional recipe-based approach; students use the same kits, and therefore instructions, as any researcher using these kits in a research laboratory. These two techniques are also cornerstone techniques in immunology being ELISA and flow cytometry for the CBA, supporting the development of student's technical skills. Once data has been collected, it is analyzed accordingly using appropriate statistical measures; all students have completed, as part of their degree, at least one unit that teaches statistics.

An important aspect of inquiry-based learning is reporting, which helps to develop a deeper understanding of the topic. A major benefit of inquiry-based learning is that it develops authentic skills, those that researchers use in their own science, and so the learning and assessment tasks associated with this learning activity are meaningful to a “real-life” laboratory situation.

Each student produces an individual practical report based on the collective data from their group, consisting of a title, an introduction, methods section, results, discussion, and reference section. Proponents of inquiry-based learning maintain that reporting is a critical aspect to learning in an inquiry-based task. Reporting has obvious educational and assessment benefits; students need to assess their data, interpret it meaningfully, and contextualize it within the current scientific literature. These aspects underlie effective scientific communication and are key components of inquiry-based writing (19). As mentioned, part of constructing this report, requires students to implement appropriate statistics in their data analysis. There is supporting evidence that integrating statistics into inquiry-based activities in the life sciences undergraduate laboratory, contributes to retention of knowledge gained and also an increase in understanding of the applications (20). Furthermore, students are encouraged to publish their work in undergraduate research journals, such as *Reinvention*, giving them scope to improve their employability, and their attractiveness as a research student.

As students are assessing multiple inflammatory markers using two different methods, it enables a more holistic understanding of an inflammatory response, which is more reflective of investigations undertaken in a research laboratory. It allows students to create a narrative and also consider the cause of any perceived incongruent results, such as an increase in the anti-inflammatory cytokine IL-10 accompanied by an increase in IL-6 which they may have only encountered in a pro-inflammatory context. They need to consider if all of their results are supported by current knowledge in the field of immunology, and if not, why this might be. It draws on their previously learned knowledge and stretches them to consider possibilities of how the immune system is reacting without resorting to the concept that they need to find a specific answer. It also reinforces the importance of the technical aspects of a study as it illustrates how accuracy, proper controls and keeping track of samples can enormously effect how well-acquired data can answer the proposed research question.

## Student and Staff Perspectives on Benefits and Drawbacks of an Inquiry-Based Task

As described, there are multiple educational benefits to an inquiry-based teaching approach. One such benefit is the genuine engagement and excitement that students and TAs demonstrate. Students are much more focused on discovering the answer to their question rather than finding out the “right” answer. TAs are co-learners in this exercise, and are equally interested in the results that their students obtain, as they also do not know the outcome. Students are involved in the authentic generation of new knowledge creating a true feeling of excitement. Students report they feel like “real scientists,” and TAs report the classes are more enjoyable, and student groups are more engaged and demonstrate better teamwork.

While engaging, it is also difficult to implement inquiry-based approaches into the curriculum. Teaching associates need good training in advance, and it requires trust to “let go” and allow students to design their intervention. Our TAs are either post-doctoral researchers or post graduate students with previous teaching experience. TAs with less teaching experience often want to be involved in the experimental design, however we encourage them to stand aside, allowing students to make mistakes, and step in at only certain stages to provide guidance. This is not to suggest that the task is not well-scaffolded, however we do encourage students to take the lead in the design of the experiment, with the TA asking pointed questions to guide students where necessary. This approach is also time-consuming, requiring more class time than a traditional laboratory class, meaning other techniques may be left out. However, we believe the skills gained in research are well worth the sacrifice of learning a new technique.

Inquiry-based learning can be intellectually draining, requiring a high cognitive load. Scholars have argued that this decreases the effectiveness of the approach (12); our approach however is highly supported through the guidance provided by TAs reducing some of the problems associated with cognitive overload. TAs provide guidance at specific stages, including at the planning and design, implementation, and analysis stages. Used in moderation, we feel inquiry-based tasks can only enhance student engagement and learning. It challenges students to apply the type of higher order thinking which is transferable to their working life, gives them a sense of the processes involved in conducting research, and therefore develops key skills required to pursue a research career. Inquiry-based learning also fosters the curiosity and creativity of our students and gives them the opportunity to experience that possibility of making new discoveries which is so integral to the scientific method and can sometimes be diminished in the more recipe-based practical classes.

Students do find the exercise challenging, but also rewarding. Anonymous student feedback about their experience include “While challenging … the inflammation prac … helped [us] work on some really useful skills, particularly for those of us looking to go into research” and another student reported that it was “the first taste of real-world science.” “It allowed for us to gain a deeper understanding of the reality of research and how much planning and thought goes into designing a study.” Staff feedback report that it allows students to develop their critical thinking skills, be creative, think about experimental design and feasibility, understand research, and helps them in future assessments that require creativity and critical thinking. While previous research has indicated that, due to the increased difficulty and cognitive-load required by the inquiry-based approach, students are quite resistant to the introduction of such tasks (3), an alternate study found that students were overwhelmingly positive about their experience (11). Similar to the latter study, we found student satisfaction in the unit being maintained since the introduction of this, and other, inquiry-based tasks, through formal student evaluations of the unit. Students report that they apply their deeper understanding in tasks that require a more in-depth appreciation of immunology and the scientific process, such as a research proposal and scientific poster which are later assessments in this, and other, immunology units.

## Final Thoughts

Inquiry-based learning is a challenging teaching tool in undergraduate teaching laboratories; it requires more time than a more traditional approach, it requires TAs to be trained differently, and to approach their teaching differently, and places a large cognitive strain on students. However, the benefits of higher student engagement and increased understanding of scientific processes outweighs the negatives. Students have a greater understanding of experimental design, the importance of controls, confounding effects, and statistics. Applying previously acquired theoretical knowledge to a genuine problem engages both students and staff, and consolidates learning. While students recognize the higher-level thinking required, and acknowledge that this is more demanding than more traditional exercises that they have completed, their high levels of engagement mean that, rather than resenting the increased difficulty, they embrace the challenge and feel more at ease in their understanding of immunology and the scientific process.

## Author Contributions

MD and KM contributed to the concept and design of the manuscript. KM and AB designed and implemented the pedagogical approach outlined herein. MD, AB, and KM contributed to the writing and editing of the manuscript.

### Conflict of Interest

The authors declare that the research was conducted in the absence of any commercial or financial relationships that could be construed as a potential conflict of interest.
